# Proposed criteria for identifying GE crop plants that pose a low or negligible risk to the environment under conditions of low-level presence in seed

**DOI:** 10.1007/s11248-015-9899-z

**Published:** 2015-08-12

**Authors:** Andrew Roberts, Flavio Finardi-Filho, Subray Hegde, Juan Kiekebusch, Gonzalo Klimpel, Mark Krieger, Martin A. Lema, Philip Macdonald, Claudia Nari, Clara Rubinstein, Bernice Slutsky, Carmen Vicien

**Affiliations:** Center for Environmental Risk Assessment, ILSI Research Foundation, Washington, DC USA; Faculty of Pharmaceutical Sciences, University of São Paulo, São Paulo, Brazil; Biotechnology Regulatory Services, Animal and Plant Health Inspection Service, USDA, Washington, DC USA; Asociación de Semilleros Argentinos, Buenos Aires, Argentina; DuPont Pioneer, Johnston, IA USA; Dow AgroSciences, Indianapolis, IN USA; MAGyP, Buenos Aires, Argentina; National University of Quilmes, Buenos Aires, Argentina; Canadian Food Inspection Agency, Toronto, Canada; Monsanto Co., St. Louis, MO USA; ILSI Argentina, Buenos Aires, Argentina; American Seed Trade Association, Alexandria, VA USA

**Keywords:** Genetically engineered plants, Low level presence, LLP, Environmental risk assessment, ERA

## Abstract

The low-level presence (LLP) of genetically engineered (GE) seeds that have been approved in the country of origin but not the country of import presents challenges for regulators in both seed importing and exporting countries, as well as for the international seed trade and the farmers who rely on it. In addition to legal, financial and regulatory challenges, such LLP situations in seed may also require an environmental risk assessment by the country of import. Such assessments have typically been informed by the national framework established to support decisions related to wide scale cultivation, and frequently do not take into account the low environmental exposure and prior regulatory history of the GE plant. In addition, such assessment processes may not be well suited to the decision-making timeframe that is necessary when dealing with an LLP situation in imported seed. In order to facilitate regulatory decision making, this paper proposes a set of scientific criteria for identifying GE crop plants that are expected to pose a low or negligible risk to the environment under LLP conditions in seed. Regulatory decision makers in some importing countries may decide to use these criteria to assist in risk analysis associated with LLP situations they are experiencing or could experience in the future, and might choose to proactively apply the criteria to identify existing GE plants with regulatory approvals in other countries that would be expected to pose low risk under conditions of LLP in seed.

## Introduction

Agriculture is a global enterprise and increasingly dependent on the movement of products across international boundaries. This includes seed, which may be produced in one country and intended for planting in another. Modern agriculture is also increasingly making use of biotechnology, in the form of genetically engineered (GE) crop plants. The use of GE crop plants is regulated in most of the world and those regulations normally require an environmental risk assessment (ERA) prior to the release of a GE crop plant into the environment for large scale or commercial cultivation. However, decisions regarding the release of GE crop plants are generally determined at the national level. This has created the potential for a GE crop plant to be widely cultivated in one or more seed exporting countries prior to its approval for use as seed intended for planting in importing countries. When small amounts of GE seed that are approved in the country of origin, but not the country of import, are found in seed, this is referred to as “low-level presence” or an LLP situation in seed. Such situations have the potential to be highly disruptive to agricultural trade of both seed and agricultural commodities.

## Background

### Regulatory frameworks and environmental risk assessment for GE crops

The first GE plants were produced in laboratories in the 1980s. The potential for use of the technology in agriculture was recognized early and countries began to prepare for the eventual commercial use of GE crops in the environment. For a variety of reasons, governments established regulatory frameworks to ensure that these products were safe for humans and the environment. Importantly, in recognition of global trade in agriculture, international efforts to harmonize the regulatory risk or safety assessment of GE crop plants began almost immediately, primarily at the Organization for Economic Cooperation and Development (OECD). By the time the first GE crop plants were introduced into production in the mid-1990s, OECD had already produced a series of scientific consensus documents providing guidance on how to conduct risk/safety assessments for GE crop plants (as well as other organisms) (OECD 1986, 1993, 1996). When the Convention on Biological Diversity was finalized in 1993, Article 19 established a working group to address the safe handling and use of “living modified organisms” (i.e. GE crop plants), with respect to the conservation and sustainable use of biodiversity. This led to the Cartagena Protocol on Biosafety, which was completed in 2000 and came into force in January 2003. At the time of this writing, 168 countries have ratified the Protocol.

The result of these developments is that GE crop plants are subject to a higher level of regulatory scrutiny than has historically been the case for agricultural commodities, including seed for planting. In almost every country in the world, the cultivation or release into the environment of a GE crop plant is prohibited until an affirmatory regulatory decision is made, and these decisions are informed by an environmental risk assessment (ERA). While ERAs are conducted in accordance with internationally harmonized concepts, decisions on the release of GE crop plants into the environment almost always reside at a national level.

### Understanding seed production, and the context for LLP in seed

Historically, farmers have set aside a portion of the grain harvest they produced to save as seed for planting in the next season. This process produces highly variable results, due to the uncertain genetics of field crops, environmental factors and the presence of other materials (e.g. weed seeds) in agricultural production areas. With the development of high yielding varieties, and the wide deployment of hybrid crops which occurred during the twentieth century, the seed saving model has been largely supplanted in much of the world by a seed production model that relies on dedicated seed production fields. Together with variety protection rules, governments and industry have developed seed certification standards that ensure the provision of genetically pure seed while limiting impurities and contaminants. Organizations like the Association of Official Seed Certifying Agencies (AOSCA) work with seed producers to help them comply with seed certification standards and OECD publishes the OECD seed schemes,^1^ which establish rules for producing certified seed that is to be moved internationally. These rules include isolation distances and other measures for assuring genetic purity, as well as a program of inspections, material control and proper documentation for maintaining the identity of certified seed.

Despite these efforts, it is generally recognized that seed for production agriculture rarely achieves 100 % varietal purity. The nature of agriculture, coupled with the biology of crop plants means that “off types,” (plants or seeds that are different than the intended variety) are inevitable, and this has been true since long before the introduction of GE crop plant varieties. Varietal purity standards being used to produce certified seed of crop plants under the OECD seed schemes typically run between 97 and 99.7 %, allowing for a small amount of “off types”. Meeting this standard has proven both achievable and adequate to assure that the end user of the seed (the farmer) is getting seed which will perform predictably in accordance with expectations for the variety intended for planting.

### LLP in seed

Ten of the 19 countries responding to an OECD questionnaire indicated that they had experienced at least one instance of LLP in association with seed or commodities that can be used as seed (OECD 2013). The responses to this questionnaire reveal a variety of different LLP situations in seed, as well as the complex legal and regulatory considerations that determine how a country will respond to a detection of LLP. Among confounding factors, the point of detection for LLP in seed differs between occurrences. Detection of LLP has occurred before the seed was exported, while the seed was in transit, during testing at the port of entry or during later testing of the crop plant grown from the seed. In all cases, regulatory officials have taken steps to exclude the unauthorized GE crop plants from the environment, but depending on the circumstances of the case different methods have been used. These include blocking the import of the seed containing the LLP, destroying crops in the field, allowing the harvest of the crop plant prior to destruction, or allowing the harvest of the crop for use in food or feed (in cases where the GE crop plant associated with the LLP situation had food/feed safety approval but not approval for cultivation in the environment) while taking steps to limit or prevent future releases into the environment. In these cases, regulators and decision makers report that they made use of environmental risk assessments and the associated information available on the characteristics of the unauthorized GE crop plant.

### Workshops on LLP, and the purpose of this paper

In December 2013, the Center for Environmental Risk Assessment (CERA) at the International Life Sciences Institute (ILSI) Research Foundation convened a workshop in Buenos Aires, Argentina to discuss the ERA of LLP in seed. The subject for consideration was scientifically valid methods for using existing information, including prior ERA(s) conducted in the country of origin or other countries, to inform assessments of risk for LLP in seed. Participants in the workshop included scientists and regulators from governments and the private sector. The proceedings of the workshop are publically available (CERA 2014). Among other things, the workshop participants concluded that LLP in seed would be best addressed by developing proactive and predictable approaches to ERA of LLP in seed. This included the possibility of pro-actively identifying GE crop plants that would be considered to pose a low risk to the environment under conditions of LLP in seed when a prior ERA has been conducted by a competent authority in another country. Such a list of GE crop plants would differ by country and the development of such a list would require both access to ERA information as well as confidence in the approach to risk assessment used in the country where the seed associated with the LLP situation originated.

To continue the work begun in Buenos Aires, a second workshop was convened in Santiago, Chile in July 2014. Over the course of 2 days, participants discussed possibilities for advancing scientific discussions related to ERA of LLP and it was determined that elaboration of criteria to identify GE crop plants that could be considered to pose low or negligible risk to the national environment under conditions of LLP would provide valuable scientific support for countries and regulators interested in this approach to prepare for LLP situations in seed.

It is fully recognized by the workshop participants and the authors of this paper that LLP in seed remains a situation to be avoided, and the development of these criteria should not be misconstrued as an attempt to normalize LLP, or to suggest particular policies or decisions in the case of an LLP incident. It is instead a recognition that detections of LLP in seed necessitate quick decisions and therefore decision makers need access to reliable information to evaluate the potential risk to the environment arising from an LLP situation. In such cases, the proactive use of risk assessment information prior to an occurrence of LLP can help regulators devote resources appropriately in order to protect the environment, minimize economic disruptions and return the situation to compliance with applicable laws and regulations.

## ERA under conditions of low exposure, and criteria for identifying GE crop plants that can be expected to pose low or negligible risk to the environment under conditions of LLP in seed

LLP in seed represents a condition of low exposure of an unauthorized GE crop plant to the environment. A previous publication has identified issues of ERA that are most important to consider under low exposure scenarios, and recommends a stepwise approach to ERA (Roberts et al. 2014). Following this approach, it is necessary to first understand the characteristics of the GE crop plant, the incorporated trait and the environment where it is introduced, and then determine the likelihood that the plant will persist or multiply in the environment, thereby increasing environmental exposure. If there is confidence that this will not occur, then a more detailed risk assessment may not be required. This stepwise approach is informative for developing criteria for identifying GE crop plants that can be predicted to pose low or negligible risk under conditions of LLP in seed. Three general criteria are proposed:

1. Experience and knowledge with the crop plant indicates that the crop will not survive, persist and multiply in the receiving environment without human intervention
2. Experience and knowledge with the incorporated trait (either the phenotype or the gene/protein) indicates that it does not pose a risk to the environment under conditions of LLP in seed
3. A previously conducted ERA concludes that the GE crop plant does not have altered characteristics with respect to growth or reproduction that would affect survival and persistence in the receiving environment

These three criteria and points to consider when applying them to identify GE crop plants that can be expected to pose low or negligible risk under conditions of LLP in seed are elaborated below.

### Experience and knowledge with the crop plant indicates that the crop will not survive, persist and multiply in the receiving environment without human intervention

Risk to the environment is the probability of realizing an adverse (unwanted) outcome based on how hazardous or potentially harmful the stressor is, considering its exposure in the environment. If there is no exposure, then there can be no risk. As exposure increases, the level of risk becomes much more dependent on the degree of hazard, or how serious the harm may be. Under conditions of LLP in seed, the exposure will be above zero, but it will be low. Low exposure correlates with low probability of harm except in cases where hazards are extreme (this will be addressed in the third criterion) (Hill 2005; OGTR 2009). Confidence that a GE crop plant poses a low risk under conditions of LLP in seed can be attained once there is a high degree of confidence that the GE crop plant will not persist in the environment or multiply, thereby increasing the level of exposure.

This confidence can be derived from the experience and knowledge of local farmers, scientists and regulators who deal with crops. This existing knowledge base [referred to as “familiarity” with the plant species (OECD 1993)] forms the basis for comparison in an ERA for a GE crop plant, and can readily inform decisions about unauthorized GE crop plants in relation to crop plants which are currently cultivated within the importing country.

Specific considerations for determining if a crop plant meets this criterion include:

- The plant has been grown as a crop in the importing country for a significant period of time and does not survive or persist in the environment without human intervention

The ability of a plant to survive and persist in the environment depends on a combination of its hardiness, growth habit, reproductive biology and the environmental conditions where it is grown. Most of these characteristics will be determined by the crop plant’s biology and the growing environment, rather than by an introduced trait in a GE crop plant. Therefore, prior experience with the non-engineered crop species in-country will provide the best evidence as to whether or not the GE crop plant is likely to survive or spread.

- The crop plant is cultivated in other countries with comparable environments and has not been shown to survive or persist in the environment without human intervention

It may be possible to conclude with confidence that a plant will not survive or persist based on experience in comparable environments from outside the national jurisdiction. For example, the ability of many plants to survive is limited by freezing conditions associated with winter. So, for example, if a plant is known to be sensitive to frost it may be possible to conclude that it will not survive the winter in a country where winter temperatures are routinely below freezing even if the plant has not been grown as a crop in the importing country.

- Specific management conditions that are relevant to the agricultural use of the crop plant limit its ability to survive and persist in the environment

Although this may be considered a subset of the first two considerations, assessors can take advantage of knowledge of how a crop is grown and managed in the environment. For example, some crops, such as alfalfa (*Medicago sativa*) are routinely harvested for hay production prior to flowering. This greatly reduces the potential for the plant to persist in the environment.

- The presence of any wild relatives of the crop plant to which gene flow may occur, leading to increased exposure or persistence of the GE trait in the environment

The presence of sexually compatible wild relatives is a complicating factor for any ERA, because there is the possibility of greater exposure to transgene products due to gene flow to wild relatives. Although the presence of a wild relative does not necessarily exclude a GE crop plant from presenting low risk under conditions of LLP, assessors should have an understanding of how likely it is that a transgene and its accompanying traits will move into wild populations.

### Experience and knowledge with the introduced trait indicates that it does not pose a risk to the environment under conditions of LLP in seed

In order to determine whether a GE crop plant poses low or negligible risk to the environment under conditions of LLP in seed, it must be understood how the trait, along with the gene and the expressed product (e.g. the protein), if any, will interact with the environment in producing the phenotype. This understanding can be derived from previous experience with the same trait in the same or similar crop plants—derived either through conventional breeding or genetic engineering. It might also be derived from a previous environmental risk assessment conducted on a similar GE crop that incorporates the same, or a similar transgene. A trait may qualify or meet this criterion if it meets any one or more of the specific considerations listed below:

- Prior assessments of the crop plant and transgene combination for cultivation have concluded that the trait is not likely to pose a risk to the environment

If the trait is the result of a transgene that has been previously assessed in the same crop in the importing country, and found to pose little risk to the environment then there is very little reason to think a different transformation event would pose a significant risk under conditions of LLP. The only potential difference will be molecular genetic differences resulting from the transformation and breeding process, and there is increasing evidence that these are not relevant for ERA (Schnell et al. 2014).

- Prior assessments of the transgene in one or more different crop plants have concluded that the trait is not likely to pose a risk to the environment

Similarly, if the transgene has been assessed in other plants, then the trait, resulting from any expressed gene products (e.g. protein, RNA), has already been determined to pose a low risk. Provided the receiving environment for the different crop plants is sufficiently similar, assessors should be able to draw conclusions from the previous assessment with regard to risk under conditions of LLP.

- Approvals or experience with similar/comparable traits in the same crop plant generated by a different transgene or introduced through alternative production methods (e.g. through traditional breeding) indicate that the trait is unlikely to pose a risk to the environment

Finally, if the same trait has been introduced into the crop plant previously, using a different transgene or alternative breeding method, then this provides evidence that the crop trait combination is unlikely to pose a risk to the environment. In particular, that the crop plant is unlikely to show increased survival and persistence. Because the product of the transgene (e.g. protein, RNA) has not been considered in the previous assessment, information on the gene product may also need to be taken into account.

### A previously conducted ERA concludes that the GE crop plant does not have altered characteristics with respect to growth or reproduction that would affect survival and persistence in the receiving environment

A case specific existing ERA, normally accomplished for unconfined release, is comparative and addresses whether the GE crop plant has any altered characteristics when compared to the non-engineered plant species. It is important to understand if the GE crop plant has altered growth, reproductive or dissemination characteristics that would influence its ability to survive and persist in the environment. This is typically done through observations related to plant growth habit that are collected during field trials. Such an ERA does not necessarily have to come from the country of export. Any ERA which meets the criteria below could be used, even if produced in a third party country.

Specific considerations for whether an existing ERA meets the criterion include:

- The assessment is transparent and accessible

In order to determine the reliability of an existing ERA, risk assessors must have access to that assessment and relevant data that inform it to ensure confidence that the conclusions of the assessment are supported by that data.

- The use of the GE crop plant considered in the existing ERA is comparable to the use in the country of import

The use of crop plants may vary between countries, and it is important to confirm that the use of the crop plant in the country which conducted the ERA is comparable to the use in the importing country where an LLP situation might develop. If the agricultural practices are similar, and the crop is used for the same purposes (e.g. food or feed) then the use of the GE crop plant considered in the ERA is likely comparable to the use in the country of import.

- The ERA considers whether the GE crop plant has altered growth, survival or reproductive characteristics when compared to the untransformed plant

A large part of the value in considering an ERA conducted by another country comes from the consideration of whether the crop plant has certain altered characteristics. This is a common feature to ERA for GE crop plants, and it gives confidence that the crop plant, under conditions of LLP in seed, can be expected to perform similarly to the non-GE counterpart with respect to its survival.

- The potential for the unauthorized GE crop plant to survive, persist or reproduce in the environment where the existing ERA was conducted is comparable to its potential to survive, persist or reproduce in the country of import

If the assessment is going to be considered informative with respect to the ability of a GE crop plant to survive and persist under LLP conditions, then it stands to reason that the assessment must be conducted in consideration of a similar environment. This doesn’t mean that the environments must be identical, but if the environmental conditions are radically different, then it’s important to consider how this might affect the results of the assessment with regard to survival and persistence of the unauthorized GE crop plant in the importing country. If the survival of the non-GE comparator is similar in the environment where the existing ERA is conducted with the survival in the importing country, then this should afford some confidence that the conclusions will be applicable.

## Using these criteria to develop list of GE plants that can be expected to pose low or negligible risk under conditions of LLP in seed

The regulation of agricultural biotechnology is primarily a national activity and countries have designed their regulatory systems to meet their national needs. The specific goals of each system vary, but they all include the desire to protect the environment and biodiversity, while taking advantage of beneficial technologies. Most countries also have a compelling interest in facilitating agricultural trade, both for the purpose of maintaining food security and to make sure that farmers have access to the best possible seed. Balancing these imperatives, and meeting regulatory and legal requirements in the face of an LLP situation in seed is a challenge. The purpose for developing these criteria is to provide a scientifically sound mechanism for identifying GE crop plants that pose a low or negligible risk to the national environment under the low environmental exposure conditions associated with LLP in seed. Using these criteria, a national authority could prepare a list of GE crop plants identified prior to any occurrence of LLP, that it is confident would pose little environmental risk under the conditions of an LLP situation in seed. There are several advantages for developing such a list, but the main one is time. For better or worse, ERA associated with GE crop plants is a time consuming process, sometimes taking years to complete, particularly for a full, commercial or unrestricted environmental release authorization. It is challenging for a regulatory authority to complete an ERA within the timeframe where it might be useful in addressing a specific instance of LLP in seed when the decision may need to be taken in a matter of days or hours, and the assessment does not begin until after the detection of the LLP situation. The development of list of pre-evaluated GE plants that may potentially present an LLP situation in seed allows regulators to conduct a proactive review of available information and determine if the GE crop plant meets the criteria for posing a low or negligible risk under LLP conditions without the time pressure of an LLP occurrence. Although the availability of the information needed to assess whether a GE plant meets the criteria presented here would be expected to greatly facilitate ERA, the resultant identification of those GE plants and the use of a list can increase the transparency and predictability, allowing immediate decisions that would otherwise be significantly delayed because of uncertainty over environmental risk.

The development of list of pre-evaluated GE plants that may potentially present an LLP situation in seed allows regulators to conduct a proactive review of available information and determine if the GE crop plant meets the criteria for posing a low or negligible risk under LLP conditions in seed without the time pressure of an LLP occurrence. While the purpose of this paper is to provide a set of criteria to identify GE plants that pose low or negligible risk under LLP situations in seed, whether countries find the development of a list *per se* useful or how such a list might be used if developed, to proactively address LLP situations is a matter of policy. Development of a list of items can be a tool for beneficial purposes, but lists can become immutable permanent features lacking flexibility, so their use should fit the purpose. Theoretically there can be several such lists possible between/among trading partners depending upon the crops, traits and the environment. Some countries may have little or no discretion when it comes to regulatory action regarding LLP in seed (OECD 2013). However, others may wish to take actions in accordance with the risk to the environment. This includes decisions on how to address the LLP situation, whether and what risk management measures are appropriate, and how to ensure future compliance with applicable laws and regulations (OECD 2013).

## Footnotes

1 http://www.oecd.org/tad/code/oecdseedschemesrulesandregulations.htm
